# Supplementary figures and images for: Tetrahydrocannabinol Reduces Hapten-Driven Mast Cell Accumulation and Persistent Tactile Sensitivity in Mouse Model of Allergen-Provoked Localized Vulvodynia
Source: Int J Mol Sci. 2019 May 1;20(9):2163. doi: 10.3390/ijms20092163 (PMC6539044; doi:10.3390/ijms20092163)

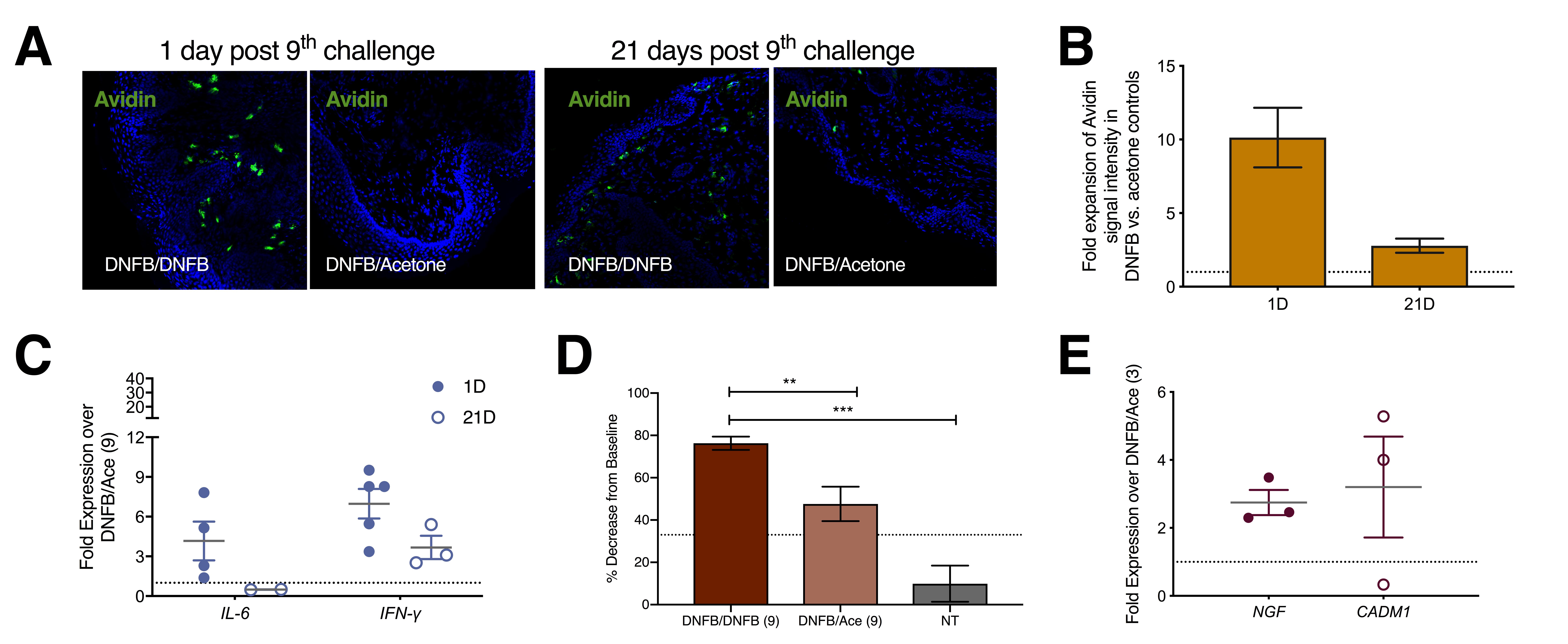

Supplement: Supplementary file 1 [file ijms-20-02163-s001.zip › Chatterjea Supplementary Figures/Figure S1.tiff]

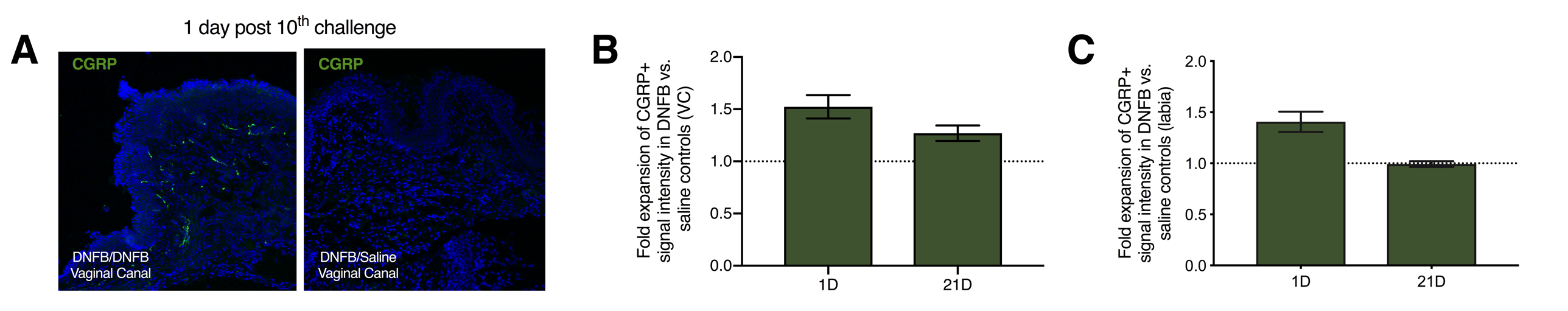

Supplement: Supplementary file 1 [file ijms-20-02163-s001.zip › Chatterjea Supplementary Figures/Figure S2.tiff]
